# Acute Kidney Injury and Risk of Adverse Neurocognitive Outcomes: A Systematic Review and Meta-Analysis

**DOI:** 10.1212/WNL.0000000000218031

**Published:** 2026-05-07

**Authors:** Dearbhla M. Kelly, Eoin M. Kelleher, Peter M. Rothwell

**Affiliations:** 1 Wolfson Centre for the Prevention of Stroke and Dementia, Nuffield Department of Clinical Neurosciences, University of Oxford, United Kingdom;; 2 Division of Critical Care, Department of Anesthesiology, Brigham and Women's Hospital, Boston, MA;; 3 Department of Anesthesia, Critical Care and Pain Medicine, Massachusetts General Hospital, Boston; and; 4 Nuffield Department of Clinical Neurosciences, University of Oxford, United Kingdom.

## Abstract

**Background and Objectives:**

Chronic kidney disease is a recognized risk factor for adverse neurocognitive outcomes, but the effect of acute kidney injury (AKI) on brain health remains less well defined. We conducted a systematic review and meta-analysis to evaluate associations between AKI and subsequent risk of stroke, delirium, and dementia.

**Methods:**

Eligible studies were identified by searching Ovid MEDLINE and Embase from inception (Ovid: January 1946; Embase: January 1970) until April 2025. Studies were included if they reported quantitative estimates with measures of precision for the association between AKI and delirium, stroke, or dementia in adult populations. Two reviewers independently screened and extracted data, and study quality was assessed using standardized criteria. Study characteristics, participant demographics, and adjusted effect estimates (hazard ratios [HRs] or odds ratios [ORs]) with 95% CIs were extracted. Pooled HRs and ORs with 95% CIs were calculated using random-effects models. Heterogeneity was evaluated with the χ^2^ test and *I*^2^ statistic, and sources of heterogeneity were explored through prespecified subgroup analyses and meta-regression.

**Results:**

We identified 49 studies comprising 11,253,825 participants with 1,279,145 events. Individuals with AKI were at increased risk of stroke (pooled adjusted HR 1.35, 95% CI 1.20–1.52), delirium (pooled adjusted OR 1.76; 1.42–2.17), and dementia (pooled adjusted HR 1.64, 1.41–1.89). A gradient of risk across increasing AKI stages was demonstrated for stroke (stage 1: HR 1.11; 1.00–1.23; combined stages 2 and 3: HR 1.57; 1.35–1.81). AKI was also associated with higher in-hospital and 90-day mortality poststroke (pooled HR 2.13, 1.56–2.90, and 4.81, 2.55–9.08, respectively) and with 90-day disability (pooled adjusted OR 1.47, 1.22–1.76). Associations between AKI and all outcomes were directionally consistent across sensitivity analyses and pooled propensity score–matched studies.

**Discussion:**

In this systematic review and meta-analysis, AKI was consistently associated with increased short-term and long-term neurocognitive risk, including stroke, delirium, and dementia. These findings suggest that AKI may identify individuals vulnerable to both acute and chronic brain injury. Further studies are needed to clarify mechanisms linking AKI to brain injury and to identify strategies to mitigate neurocognitive risk in this high-risk population.

## Introduction

Acute kidney injury (AKI) is a major and growing global health concern, affecting approximately 1 in 5 hospitalized adults and up to two-thirds of critically ill patients.^1^ Beyond its immediate consequences, AKI has been consistently linked to increased long-term risks of mortality, progression to chronic kidney disease (CKD), and increased health care resource utilization.^2^

The brain-kidney axis has been well described in CKD, where reduced kidney function is strongly associated with increased stroke risk, cerebral small vessel disease, vascular cognitive impairment, and dementia.^3^ In contrast, the neurologic consequences of AKI remain comparatively underexplored. Emerging epidemiologic evidence suggests that AKI may be associated with increased risk of delirium, stroke, and dementia, with risk escalating in relation to kidney injury severity.^4-6^ However, these associations may be confounded by factors such as age, vascular multimorbidity, preexisting CKD, cognitive impairment, frailty, and socioeconomic factors. Moreover, the existing literature has not been systematically synthesized to quantify the magnitude and consistency of these associations.

Biological mechanisms plausibly linking AKI to acute and chronic brain injury include systemic inflammation, endothelial dysfunction, blood–brain barrier disruption, and uremic toxin accumulation.^7,8^ These processes may promote both acute encephalopathy and longer-term cerebrovascular and neurodegenerative changes.

Neurocognitive disorders are among the leading contributors to disability, reduced quality of life, and health care costs worldwide.^9^ Understanding whether AKI contributes to their incidence is, therefore, of substantial clinical and public health importance.

In this systematic review and meta-analysis, we aimed to evaluate the effect of AKI on risk of stroke, delirium, and dementia.

## Methods

### Data Sources and Searches

The study protocol was registered prospectively on PROSPERO (CRD420251018453) and reporting followed Preferred Reporting Items for Systematic Reviews and Meta-Analyses guidelines.^10^ We searched MEDLINE and EMBASE databases (from inception to April 2025) using a search strategy developed in collaboration with a specialized librarian that combined text word and medical subject headings without language restrictions (eTable 1). Unpublished studies, including gray literature (e.g., preprints, conference abstracts, and trial registries), and commentaries were not included.

### Study Selection

Studies were included if they reported quantitative estimates with a measure of precision of the association between AKI and at least one of the outcomes of interest in an adult population (age ≥18 years), used consensus definitions of exposure, and included a non-AKI comparator arm. AKI was defined according to Risk, Injury, Failure, Loss of kidney Function, and End-stage kidney disease (RIFLE), Acute Kidney Injury Network (AKIN), and Kidney Disease: Improving Global Outcomes (KIDGO) criteria using serum creatinine and/or urine output (eTable 2). Exclusion criteria included studies that did not describe their method of exposure ascertainment, studies based in pediatric populations, case reports/series, and non–peer-reviewed literature. The outcome measures were delirium, dementia, or stroke. Collectively, these outcomes are referred to as adverse neurocognitive events.

### Data Abstraction and Quality Assessment

Two independent reviewers (D.M.K. and E.M.K.) performed the initial search and study selection. All potentially eligible citations were screened by title and abstract. The full text of each potentially relevant study was independently assessed in detail by 2 reviewers for inclusion in the review. Disagreements were managed through discussion; if consensus could not be reached, a third reviewer (P.M.R.) was consulted. When multiple publications arose from the same study cohort, the most recent and complete dataset was prioritized to avoid duplicate inclusion of participants.

Data were independently extracted by 2 reviewers using piloted forms. The following properties were extracted from each study: study characteristics (year of publication, location, design, setting, number of patients, duration of follow-up, definition and method of AKI ascertainment, definition and method of outcome [delirium, dementia, stroke] ascertainment), patient demographic characteristics (age, sex, and race), comorbidity (preexisting CKD, baseline estimated glomerular filtration rate (eGFR), preexisting cognitive impairment, diabetes mellitus, hypertension, known vascular diseases, chronic heart failure, chronic liver disease, and malignant tumor), use and modality of dialysis, and illness severity (Acute Physiology and Chronic Health Evaluation score, mechanical ventilation, and vasopressor use). Comorbidities were recorded as defined by the investigators. We also noted if participants were recruited at a time of potentially higher neurocognitive risk including around an acute coronary event, coronary revascularization procedure, or carotid arterial intervention. Risk estimates (odds ratio [OR], risk ratio, or hazard ratio [HR]) for delirium, dementia, and stroke were also extracted. We obtained effect estimates from the most fully adjusted model presented noting which variables the model had adjusted for. The standard error of the estimate was also extracted or estimated from the reported 95% CI.

Study quality and risk of bias were independently adjudicated by 2 reviewers using the Newcastle-Ottawa Scale.^11^ Discrepancies were resolved through discussion or by a third reviewer if consensus could not be reached. Publication bias was assessed using the Egger test for funnel plot asymmetry.

### Statistical Analysis

We converted ORs and HRs associated with the presence or categories of AKI to their natural logarithms and synthesized log ORs and HRs and standard errors using the DerSimonian and Laird method in a random effects model. All *p* values were 2-sided, and results were considered statistically significant at the 0.05 level. When studies published more than 1 estimate of the association between AKI and risk of neurocognitive outcomes (e.g., by AKI severity), a within-study summary estimate was obtained using a fixed effects model. Heterogeneity among included studies was evaluated by χ^2^ statistics and the *I*^2^ test. Following Cochrane guidance, *I*^2^ values below 40% were interpreted as indicating minimal heterogeneity, whereas values exceeding 75% were considered to reflect substantial heterogeneity.^12^ We used subgroup, sensitivity, and meta-regression analyses to explore sources of inconsistency and heterogeneity. Subgroups were prespecified and included study characteristics (study design, size, location, duration of follow-up, AKI definition) and participant characteristics (age, sex, race, AKI stage, prevalence of diabetes, hypertension, CKD, preexisting cognitive impairment, clinical setting [intensive care unit (ICU), angiography, cardiovascular surgery]). Angiography included patients undergoing limb or coronary angiography or transcatheter aortic valve replacement. We also prespecified a subgroup according to the method of adjustment for potential confounders (propensity scoring methods vs other). For studies using propensity score methods, details regarding covariates included, analytic approach (matching or weighting), matching parameters, and covariate balance assessment are summarized in eTable 3. Sensitivity analyses included elimination of studies at high risk of bias, of retrospective design, or of inadequate follow-up or those with an average baseline eGFR of <60 mL/min per 1.73 m^2^. Additional sensitivity analyses included the exclusion of studies with extreme outlier risk estimates and a leave-one-out analysis to assess the robustness of pooled estimates. Univariable meta-regression analyses were performed to examine for evidence of effect modification on the association between AKI and outcomes. These analyses considered the following study-level demographic and clinical variables where sufficient data were available: publication year, duration of follow-up, study size, patient age, baseline eGFR, proportion of patients who were male or had a history of diabetes, hypertension, vascular disease, pre-existing CKD or cognitive disorders, and prevalence of kidney replacement therapy (KRT) use. For all analyses, we used R version 4.3.2 (R Foundation for Statistical Computing, Vienna, Austria).

### Standard Protocol Approvals, Registrations, and Patient Consents

This study is a systematic review and meta-analysis of previously published data and did not involve direct contact with human participants; therefore, institutional review board approval and patient consent were not required. The protocol was registered prospectively on PROSPERO (CRD420251018453).

### Data Availability

Data were derived from previously published studies identified through systematic database searches. The data extracted and code used for analyses are available from the corresponding author upon reasonable request.

## Results

### Characteristics of the Included Studies

**Figure 1:**
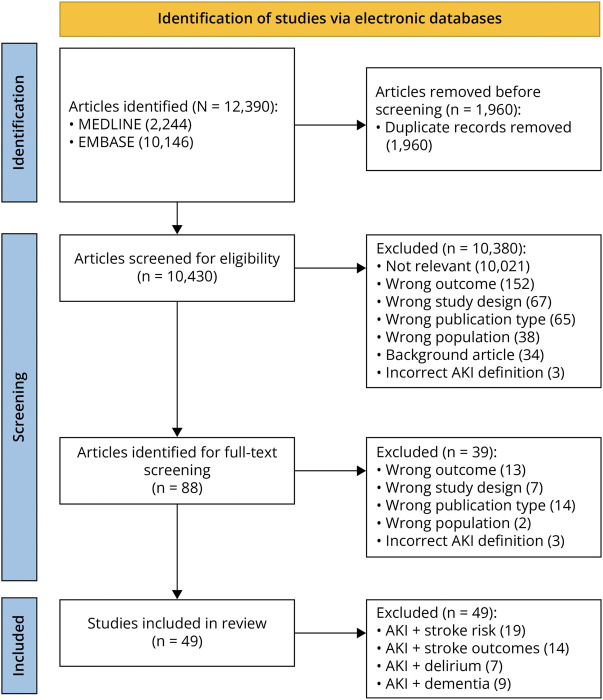
Identification and Inclusion of Study Reports of AKI and Neurocognitive Risk AKI = acute kidney injury.

The systematic review identified 10,430 unique citations, of which 88 articles were retrieved for full-text review after being screened by title and abstract (Figure 1). Forty-nine studies were eligible for inclusion (eTable 3), including 11,253,825 patients, of whom 1,279,145 (11.4%) had AKI. The mean or median patient age ranged from 47.5 to 83.8 years, and male participants accounted for between 41.9% and 79.0% of the patient cohorts. Data were extractable for the association between AKI and stroke, stroke outcomes, delirium, and dementia from 19, 14, 7, and 8 studies, respectively.

The data were derived from 37 retrospective cohort studies, 10 prospective cohort studies, and 2 randomized controlled trials. Characteristics of the included studies and randomized trials are described in eTables 3 and 4. The median sample size was 2,119 patients (range 214–7,068,334 patients). The median duration of follow-up was 1 year (range 0.01–15.5 years). AKI was most commonly diagnosed based on KDIGO criteria (26 studies; 53.1%) followed by International Classification of Diseases (ICD) coding (10 studies; 20.4%), AKIN (7 studies; 14.3%), and then RIFLE (2 studies; 4.1%). Most studies were performed in Europe (n = 19; 38.8%) followed by North America (n = 15; 30.6%) and Asia (n = 12; 24.5%). Nine studies (18.4%) were in the setting of either cardiac surgery or percutaneous cardiovascular interventions, and 5 studies (10.2%) occurred in the setting of noncardiac surgery.

### Stroke Risk

**Figure 2:**
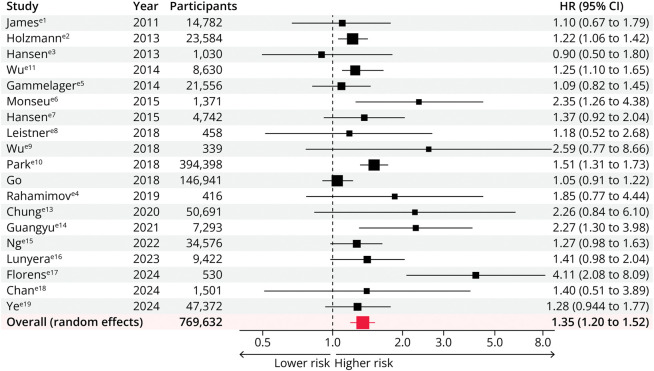
Association Between Acute Kidney Injury and Risk of Stroke HR = hazard ratio.

Nineteen studies including 769,632 participants examined the risk of stroke after an episode of AKI.^e1-e19^ The risk of stroke was 35% higher in patients with AKI than in patients without AKI (pooled adjusted HR 1.35; 95% CI 1.20–1.52) (Figure 2). Although there was moderate heterogeneity between studies (*I*^2^ = 53.04%; *p* = 0.0035), there was no qualitative difference in risk. In studies that reported the risk of stroke by AKI stage, the risk increased from stage 1 (pooled adjusted HR 1.11; 95% CI 1.00–1.23) to stage 2/3 (pooled adjusted HR 1.57; 95% CI 1.35–1.81).

An AKI event was associated with an increased risk of subsequent stroke in all subgroups when estimates were stratified by study design, size, location, setting, duration of follow-up, mean age groups, sex, prevalence of diabetes mellitus, hypertension, and CKD, proportion receiving KRT, setting, and use of propensity score matching (eFigure 1). There were stronger associations with stroke risk in prospective cohort studies (pooled adjusted HR 3.06 [1.77–5.28]; *p* for interaction <0.001), in studies with longer duration of follow-up (5–10 years; pooled adjusted HR 2.72 [1.74–4.24]; *p* for interaction <0.001), in studies with a higher prevalence of hypertension (pooled adjusted HR 1.82 [1.29–2.56]; *p* for interaction = 0.046), and CKD (pooled HR 2.90 [1.33–6.30]; *p* for interaction = 0.002).

Although the association also appeared greater in smaller studies (<2,500 participants; pooled adjusted HR 1.66 [1.15–2.39]; *p* for interaction = 0.649), in those with higher prevalence of diabetes mellitus (pooled adjusted HR 2.35 [1.26–4.38]; *p* for interaction = 0.265), and in studies set after noncardiac surgery (pooled adjusted HR 2.27 [1.39–3.69]; *p* for interaction = 0.539), these differences were not statistically significant. The association was also preserved in studies restricted to those with propensity score matching (pooled adjusted HR 1.27 [1.09–1.48]; *p* for interaction = 0.309). Subgroup analyses did not show evidence of effect modification by any other study or participant characteristics including AKI definition (*p* for interaction = 0.534) and across categories of KRT use (*p* for interaction = 0.397) (eFigure 1).

**Figure 3:**
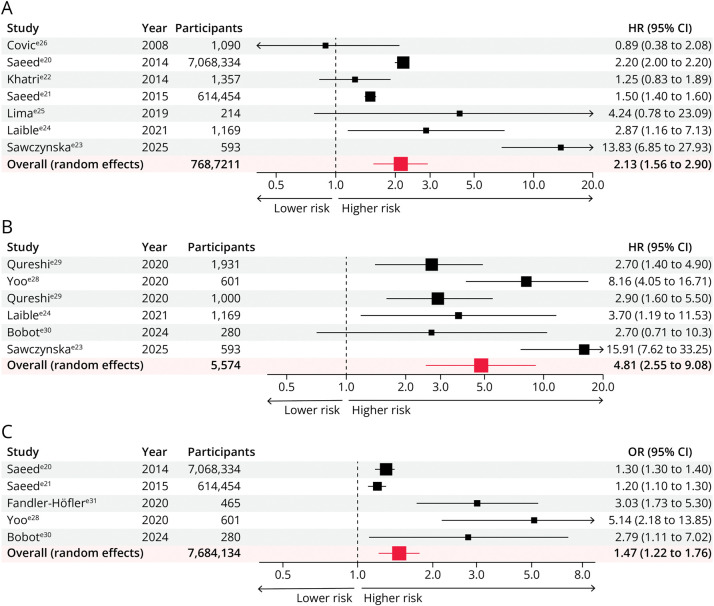
(A) Association Between Acute Kidney Injury and Risk of Hospital Mortality, (B) 90-Day Mortality, and (C) Poor Functional Outcome at 90 Days in Patients With Acute Stroke HR = hazard ratio; OR = odds ratio.

Across 7 studies including 7,687,211 participants,^e20-e26^ AKI was associated with higher in-hospital mortality poststroke (pooled HR 2.13, 95% CI 1.56–2.90), but considerable heterogeneity was present (*I*^2^ = 95.12%; *p* < 0.0001) (Figure 3A). Six studies (5,574 participants) reported 90-day mortality,^e23,e24,e27-e30^ showing substantially increased risk post-AKI (pooled HR 4.81, 95% CI 2.55–9.08; *I*^2^ = 73.72%; *p* = 0.002) (Figure 3B). Five studies (7,684,134 participants) assessed 90-day disability,^e20,e21,e28,e30,e31^ with AKI associated with higher odds of disability (pooled OR 1.47, 95% CI 1.22–1.76; *I*^2^ = 82.80%; *p* = 0.0001) (Figure 3C).

### Delirium Risk

**Figure 4:**
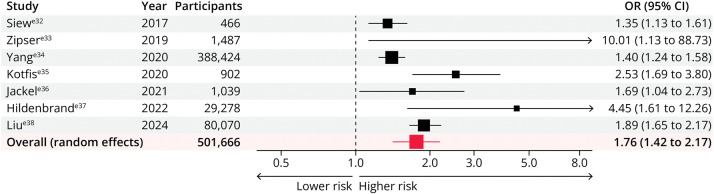
Association Between Acute Kidney Injury and Risk of Delirium OR = odds ratio.

The development of delirium after AKI was examined by 7 studies, which included 501,666 participants.^e32-e38^ Patients who developed AKI were at a significantly higher risk of delirium than were patients without AKI (pooled adjusted OR 1.76; 95% CI 1.42–2.17), with substantial heterogeneity between studies (*I*^2^ = 76.41%; *p* = 0.0003) (Figure 4). The risk of delirium increased with higher AKI severity: pooled adjusted OR 1.51 (95% CI 0.80–2.84) for stage 1 AKI; pooled adjusted OR 1.72 (95% CI 1.14–2.58) for stage 2 AKI; and pooled adjusted OR 3.58 (95% CI 1.38–9.29) for stage 3 AKI (eFigure 2).

AKI was associated with increased risk of delirium across all subgroups (eFigure 3). The association was stronger in European studies (pooled adjusted OR 2.51 [1.59–3.96]; *p* for interaction = 0.040) and in those of gastroenterology, hepatology, or neurology inpatients (pooled adjusted OR 5.14 [2.05–12.90]; *p* for interaction = 0.041). However, the association was not significant when restricted to prospective studies only (pooled adjusted OR 2.91 [0.92–9.15]; *p* for interaction = 0.985), to studies of younger populations (55–64 years; pooled adjusted OR 2.91 [0.92–9.15]; *p* for interaction = 0.985), or in studies where the preexisting prevalence of prior cognitive disorders was greater than 2% (pooled adjusted OR 2.95 [0.59–14.89]; *p* for interaction = 0.937). The association between AKI and delirium did not differ by AKI definition (*p* for interaction = 0.766).

### Dementia Risk

**Figure 5:**
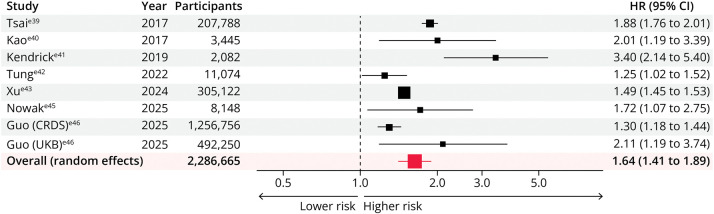
Association Between Acute Kidney Injury and Risk of Dementia CRDS = China Renal Data System; HR = hazard ratio; UKB = UK Biobank.

Nine studies including 2,288,203 participants reported the long-term risk of dementia associated with AKI; however, only 8 studies reported results suitable for pooling.^e39-e46^ The study by Guo et al.^13^ was counted as 2 separate studies in this analysis, as it reported independent results from the China Renal Data System and the UK Biobank cohorts. Across all dementia studies, prevalent dementia was excluded at cohort entry, with additional exclusion of antidementia drug use in selected cohorts^5,13^ and adjudicated exclusion of both mild cognitive impairment and dementia in the Systolic Blood Pressure Intervention Trial (SPRINT) study.^14^ An episode of AKI was associated with an increased risk of subsequent dementia (pooled adjusted HR 1.64 [1.41–1.89]) (Figure 5), with considerable heterogeneity between studies (*I*^2^ = 89.83%; *p* < 0.0001). Although this association was consistent across all subgroups, it was stronger in smaller studies (<2,500 participants; pooled adjusted HR 3.40 [2.14–5.40]; *p* for interaction = 0.032) (eFigure 4). However, the effect size and direction were preserved in studies restricted to those with propensity score matching (pooled adjusted HR 1.75 [1.37–2.24]; *p* for interaction = 0.644), in those with larger populations (≥20,000; pooled adjusted HR 1.58 [1.33–2.00]), and in those with lower baseline comorbidity (diabetes mellitus <25%: 1.47 [1.30–1.65]; hypertension <25%: 2.05 [1.40–3.02]; CKD <25%: 1.63 [1.40–1.90]). The association was also consistent across various AKI definitions (*p* for interaction = 0.784).

### Sensitivity Analyses

Prespecified sensitivity analyses were performed to confirm the robustness of findings across variations in methodology. There were no qualitative differences in the pooled estimates of risk after exclusion of studies that were retrospective, had insufficient follow-up, or in which baseline eGFR was <60 mL/min per 1.73 m^2^. Overall, estimates were not heavily influenced by any single study as illustrated in the leave-one-out analysis plot (eFigures 5–7).

When extreme outliers were excluded, the association between an AKI event and risk of stroke remained largely unchanged (pooled adjusted HR 1.30 [1.18–1.43]). When studies where the average baseline eGFR <60 were excluded, the pooled adjusted HR slightly attenuated to 1.28 (1.14–1.43) but remained significant.

Although there were no qualitative differences in findings, the magnitude of the point estimate for delirium was slightly lower than that in the primary analysis when studies of lower quality and extreme outliers were excluded (pooled adjusted OR 1.61; 95% CI 1.28–2.02 and 1.65, 1.36–2.01), respectively.

Similarly, there was little diminution of the association between an AKI episode and risk of dementia when extreme outlier studies were excluded (pooled adjusted HR 1.55, 1.34–1.78).

### Meta-Regression

Sources of heterogeneity between studies were explored by meta-regression. The only study level covariate that may explain some of the difference in risk estimates for studies examining the association between AKI and stroke was duration of follow-up with studies with longer follow-up reporting greater associations (5–10 years; pooled adjusted HR 2.72 [1.74–4.24]) compared with studies with shorter follow-up (pooled adjusted HR 1.39 [0.94–2.06] and 1.05 [0.91–1.22] for ≤1 month and 1 month to 1 year, respectively; *p* for trend = 0.048). No other study-level covariate including size, age, prevalence of diabetes, hypertension, CKD, or KRT use contributed significantly to heterogeneity (eFigure 8). Similarly, meta-regression did not reveal any sources of significant heterogeneity between studies of AKI, delirium, or dementia risk (eFigures 9 and 10).

### Small Study Effects

The funnel plots for studies of AKI with subsequent stroke and delirium risk showed some asymmetry consistent with publication bias with smaller studies showing an exaggerated stroke risk association with AKI (eFigures 11 and 12). Egger test confirmed the presence of small study effects (*p* = 0.017 and 0.008, respectively). There was no evidence of small study effects on visual inspection for funnel asymmetry and using the Egger test for studies of AKI and dementia risk (*p* = 0.058; eFigure 13).

## Discussion

In this systematic review and meta-analysis including more than 11 million participants across 49 studies, AKI was consistently associated with adverse neurologic outcomes. Specifically, AKI was linked with a significantly increased risk of stroke, higher poststroke mortality and disability, and greater risk of delirium and dementia. Although heterogeneity between studies was high, no qualitative differences were identified, and the findings were robust across sensitivity analyses.

AKI was associated with a 35% higher risk of subsequent stroke, with evidence of a dose-response relationship whereby greater AKI severity conferred progressively higher risk. It is important that heterogeneity between studies was only moderate, and the association was preserved in both propensity score–matched analyses and in prospective cohorts, supporting the robustness of the finding. Stroke risk was evident even within weeks of AKI but was strongest in studies with longer follow-up, suggesting that AKI may identify patients with persistent vascular vulnerability. Whether this gradient reflects shared baseline risk (e.g., older, multimorbid populations) or whether AKI contributes to long-term vascular dysfunction remains uncertain. The risk was accentuated among individuals with hypertension, diabetes, and CKD—groups already predisposed to both AKI and cerebrovascular disease.^15^ Biological mechanisms such as systemic inflammation, endothelial injury, and prothrombotic activation after AKI, and cerebral hemodynamic disturbances during KRT, may further contribute to cerebrovascular injury.^16-18^

AKI also portended worse outcomes after stroke. Patients with prior AKI had more than double the risk of in-hospital mortality and nearly fivefold higher 90-day mortality after stroke, alongside greater odds of poststroke disability. These findings suggest that AKI may not only increase stroke susceptibility but also impair physiologic reserve and limit recovery potential once a stroke occurs. Analogous associations are well described in CKD,^19^ where altered cerebrovascular autoregulation,^20^ heightened systemic inflammation,^21^ and reduced responsiveness to acute therapies^22,23^ have been shown to worsen outcomes. It is also possible that AKI requiring dialysis may contribute to acute brain injury and reduce cerebral resilience. Using intradialytic MRI and spectroscopy, it has been demonstrated that a single hemodialysis session can induce acute brain injury, with diffusion changes in multiple white matter tracts consistent with cytotoxic edema and ischemia.^24^ Concurrent reductions in N-acetyl aspartate and choline suggest metabolic stress and impaired perfusion during dialysis.

AKI was associated with nearly a twofold increased risk of delirium, with a graded relationship between AKI severity and delirium incidence, emphasizing the interplay between acute kidney dysfunction and acute brain vulnerability across a range of neurocognitive conditions. Potential mechanisms include accumulation of neurotoxic metabolites, impaired clearance of sedative and neuroactive drugs, and systemic inflammation disrupting the blood-brain barrier.^25^

Uremic toxins such as guanidino compounds can alter neurotransmission by inhibiting gamma-aminobutyric acid and activating NMDA receptors, producing neuronal hyperexcitability and hippocampal injury.^26^ In parallel, commonly used ICU drugs such as benzodiazepines, opioids, and cefepime may accumulate in AKI and exacerbate cognitive dysfunction.^27,28^ Cytokine surges (interleukin-1, interleukin-6, tumor necrosis factor-α) further amplify neuroinflammation and microglial activation.^7^ However, there was substantial heterogeneity. Attenuation of the association in prospective cohorts and among patients with preexisting cognitive impairment may reflect more complete adjustment for frailty and baseline neurocognitive vulnerability, and potential ceiling effects in populations already at high delirium risk. The stronger association in hepatology and neurology inpatients implies that AKI may exacerbate preexisting susceptibilities, such as ammonia-related neurotoxicity in cirrhosis or blood-brain barrier dysfunction after stroke.^7,29^ Overall, these data indicate that AKI may act as a marker of increased delirium risk, emphasizing the need for proactive cognitive monitoring during and after AKI episodes, particularly in high-risk inpatient populations.

AKI was also associated with a 64% higher risk of developing dementia. Although heterogeneity was high, meta-regression did not identify explanatory study-level factors, suggesting that variability may reflect the limited number of available studies rather than true inconsistency. The association was preserved in large, prospective, and propensity score–matched studies, supporting its robustness. Although shared risk factors such as age and vascular comorbidity likely contribute, consistent effects across age groups argue against age alone as an explanation.

AKI may represent a sentinel event associated with accelerated neurodegeneration. Associations across multiple dementia subtypes—including Alzheimer disease, vascular dementia, and Lewy body–related dementias—support a broad pathophysiologic link.^5^ Experimental data demonstrate hippocampal neuronal injury, glial activation, systemic and cerebral inflammation, and blood-brain barrier disruption within 24 hours of ischemic AKI in animal models.^7^ These effects were not replicated after acute liver injury, suggesting a degree of specificity of AKI in its association with cerebrovascular and neuroinflammatory injury. Reduced kidney function has also been linked to higher levels of dementia-related biomarkers such as neurofilament light chain and phosphorylated tau 181, consistent with subclinical neuronal injury; however, longitudinal data suggest that such elevations in CKD may not reliably predict future dementia risk and may reflect altered biomarker clearance rather than Alzheimer disease–specific neuropathology.^30^ Although residual confounding and collider bias remain possible, the preservation of the association in propensity-matched studies, large cohorts, and younger populations makes it unlikely that bias alone accounts for the findings.

This review provides a systematic and wide-ranging synthesis of the evidence on the associations of AKI with stroke, delirium, and dementia risk. Strengths include a broad and sensitive search strategy, rigorous study quality assessment, and inclusion of both short-term and long-term neurologic sequelae. The analysis incorporated >11 million participants across 49 studies conducted on 5 continents and in diverse clinical settings, enhancing generalizability. The rigorous methodology was further supported by the consistency of observed associations across sensitivity analyses and subgroups. Dose-response relationships with AKI severity lend biological plausibility.

This study also has limitations. First, most included studies were observational, limiting causal inference and leaving the possibility of residual confounding. Second, statistical heterogeneity was significant, and although subgroup and meta-regression analyses did not reveal qualitative differences in risk, unexplained variance remains. Third, few included studies reported stroke outcomes by subtype precluding meaningful subtype-specific meta-analysis. Given the distinct pathophysiology and risk profiles of stroke subtypes, future studies should specifically evaluate whether associations between AKI and stroke differ for ischemic vs hemorrhagic events. Fourth, reliance on study-level rather than individual patient-level data limited our ability to apply uniform adjustments for confounders, which may have led to overestimation of risk. Although all dementia studies excluded participants with diagnosed dementia at baseline, preexisting mild or subclinical cognitive impairment was not systematically assessed outside of the SPRINT cohort,^14^ and residual confounding by baseline cognitive vulnerability cannot be fully excluded. Fifth, variable definitions of AKI (KDIGO, RIFLE, AKIN, ICD coding) and heterogeneity in ascertainment of delirium and dementia could have introduced misclassification bias; however, subgroup analyses demonstrated consistent associations across AKI definitions with no significant interaction by ascertainment method. Delirium and dementia were assessed using heterogeneous methods across studies, including clinical screening tools, specialist diagnoses, administrative ICD codes, and registry data, likely contributing to quantitative heterogeneity and outcome misclassification. Reliance on administrative coding may underestimate true event rates, and screening tools differ in sensitivity across clinical settings. Such nondifferential misclassification would be expected to bias effect estimates toward the null, suggesting that the observed associations may underestimate the true magnitude of risk. Sixth, many included studies were conducted in high-risk clinical settings, including postcardiac and noncardiac surgery, coronary intervention, ICU, and extracorporeal membrane oxygenation populations, which may limit generalizability to lower-acuity general inpatient or community populations. However, these scenarios represent common real-world contexts in which AKI occurs and in which neurologic vulnerability is clinically relevant. It is important that study setting was not a significant source of heterogeneity for stroke or dementia outcomes, and associations were consistent across surgical, medical, and critical care cohorts. For delirium, stronger associations were observed in gastroenterology/hepatology and neurology inpatients, suggesting that AKI may amplify preexisting organ-specific or disease-specific vulnerability. Nonetheless, future studies in broader general inpatient populations are needed to further define population-level risk. Finally, small study effects and possible publication bias were evident for the AKI-stroke and AKI-delirium analyses, which may have modestly inflated effect estimates.

In this systematic review and meta-analysis, AKI was consistently associated with adverse neurocognitive outcomes, including increased risk of stroke, delirium, and dementia, across diverse populations and clinical settings. These findings suggest that AKI is not only a marker of acute illness severity but may also signal vulnerability to both short-term and long-term brain injury. Future studies should focus on mechanistic pathways linking AKI to neurovascular and neurodegenerative processes—particularly the role of inflammation, blood-brain barrier disruption, and KRT. Parallel efforts to develop risk prediction tools could enable early identification of high-risk patients and inform strategies to monitor and mitigate brain health risks after AKI.

## Glossary

AKI: acute kidney injury
AKIN: Acute Kidney Injury Network
CKD: chronic kidney disease
HR: hazard ratio
ICU: intensive care unit
KIDGO: Kidney Disease: Improving Global Outcomes
KRT: kidney replacement therapy
OR: odds ratio
RIFLE: Risk, Injury, Failure, Loss of kidney Function, and End-stage kidney disease
